# Comparative Genome Analysis of *Megasphaera* sp. Reveals Niche Specialization and Its Potential Role in the Human Gut

**DOI:** 10.1371/journal.pone.0079353

**Published:** 2013-11-18

**Authors:** Sudarshan Anand Shetty, Nachiket Prakash Marathe, Vikram Lanjekar, Dilip Ranade, Yogesh S. Shouche

**Affiliations:** 1 Microbial Culture Collection, National Centre for Cell Science, Pune, Maharashtra, India; 2 Agharkar Research Institute, Pune, Maharashtra, India; Charité, Campus Benjamin Franklin, Germany

## Abstract

With increasing number of novel bacteria being isolated from the human gut ecosystem, there is a greater need to study their role in the gut ecosystem and their effect on the host health. In the present study, we carried out *in silico* genome-wide analysis of two novel *Megasphaera* sp. isolates NM10 (DSM25563) and BL7 (DSM25562), isolated from feces of two healthy individuals and validated the key features by *in vitro* studies. The analysis revealed the general metabolic potential, adaptive features and the potential effects of these isolates on the host. The comparative genome analysis of the two human gut isolates NM10 and BL7 with ruminal isolate *Megasphaera elsdenii* (DSM20460) highlighted the differential adaptive features for their survival in human gut. The key findings include features like bile resistance, presence of various sensory and regulatory systems, stress response systems, membrane transporters and resistance to antibiotics. Comparison of the “glycobiome” based on the genomes of the ruminal isolate with the human gut isolates NM10 and BL revealed the presence of diverse and unique sets of Carbohydrate-Active enzymes (CAZymes) amongst these isolates, with a higher collection of CAZymes in the human gut isolates. This could be attributed to the difference in host diet and thereby the environment, consequently suggesting host specific adaptation in these isolates. *In silico* analysis of metabolic potential predicted the ability of these isolates to produce important metabolites like short chain fatty acids (butyrate, acetate, formate, and caproate), vitamins and essential amino acids, which was further validated by *in vitro* experiments. The ability of these isolates to produce important metabolites advocates for a potential healthy influence on the host. Further *in vivo* studies including transcriptomic and proteomic analysis will be required for better understanding the role and impact of these *Megasphaera* sp. isolates NM10 and BL7 on the human host.

## Introduction

The human gut microbiome is a complex ecological niche and the interaction of this microbiome with its host is an important factor contributing towards the health status of the host [1]–[3]. Studies based on 16S rRNA gene amplicon sequencing, using next generation sequencing technologies have successfully established the relationship of the human gut microbiome with the health and disease conditions of the host [3], [4]. The representation of various bacteria through 16S rRNA gene does give insights into ‘who are present?’ but the question ‘who does what?’ remains obscure. Recent efforts have been directed towards exploring the gene content of the human microbiome using shotgun metagenomics. These studies have helped in unraveling the complex gene repertoire, which exists within the human gut. Genes coding for central metabolic pathways, production of amino acids, biosynthesis of vitamins and cofactors, degradation of xenobiotic compounds, etc., are reported to be the major genes in this complex gene repertoire [5]. In addition, efforts have been made to sequence genomes of all available isolates of human origin, which are expected to be between 1000 and 1,150 bacterial species [6]. Genomic studies give an opportunity to unravel the underlying genetic potential of the bacteria to encode a given protein and help in assigning putative adaptive features as well as functional role for a particular bacterium in an ecosystem.

The human gut microbiota is dominated by phylum *Firmicutes* and *Bacteroidetes* [3], [4]. Genus *Megasphaera* is a member of the phylum *Firmicutes*, it belongs to the class Negativicutes and comprises of Gram-negative coccoid shaped obligate anaerobic bacteria. This genus till date includes 5 validly published species (*M. cerevisiae, M. elsdenii, M. micronuciformis, M. paucivorans* and *M. sueciensis*) that have been isolated from various sources such as rumen, spoiled beer, human clinical specimens [7]–[9]. Studies on *Megasphaera* sp. from the rumen have suggested that it is an important member of the rumen microbiome, having beneficial effects on the host [10]. On the other hand, there are no studies reporting the role of *Megasphaera* sp. in the human gut.

As a part of our larger culturomics study on the Indian gut microbiota, we have isolated two potential novel bacteria belonging to the genus *Megasphaera* (isolate NM10 and BL7). In the present study, we carried out genome sequencing of these isolates in order to identify the adaptive features and to determine their gene repertoire. These isolates were the closest phylogenetic neighbors of *Megasphaera elsdenii* DSM20460, which was previously isolated from rumen. Comparative genome analysis of the genomes of the human gut isolates and the publicly available genome of ruminal isolate revealed the differential adaptive features of *Megasphaera* sp. NM10 and BL7 that are crucial for the survival in the human gut [11]. In addition, the *in silico* genome wide analysis and *in vitro* experiments revealed metabolic traits that suggest a potential beneficial effect of *Megasphaera* sp. on the human health.

## Results and Discussion

### Isolates Used in the Study

**Figure 1:**
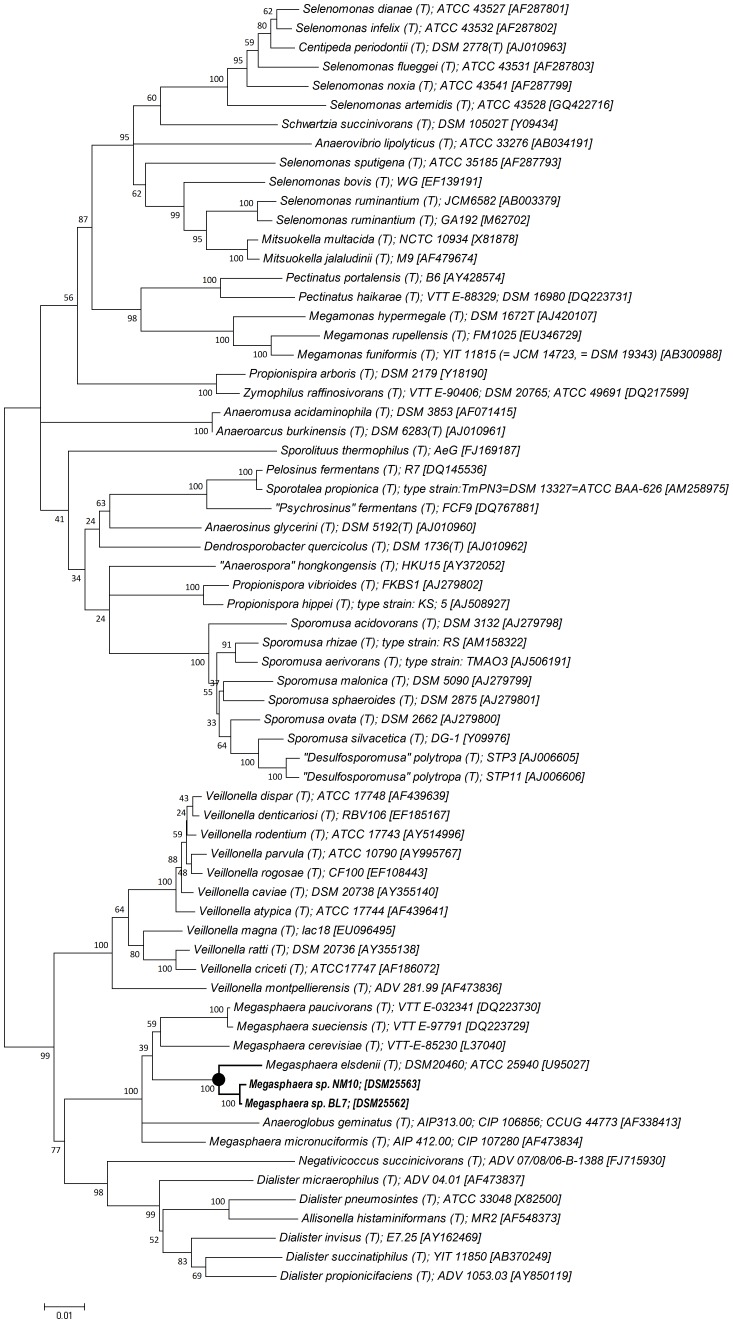
Phylogenetic tree of family *Veillonellaceae* based on 16S rRNA gene. The phylogenetic tree was constructed in MEGA4 using neighbor-joining method. The bootstrap values (expressed as percentages of 1000 replications) are shown at branch points. The scale bar represents genetic distance (1 substitution per 100 nucleotides). Isolates in present study are in bold.

The two isolates of *Megasphaera* sp. NM10, BL7 were isolated from the feces of two healthy Indian individuals. The strain BL7 was reported in our previous study and the strain NM10 was isolated as a part of our larger culturomics study on gut microbiota of the Indian individuals [12]. Institutional ethical clearance (NCCS, Pune, India) and informed consent was obtained from the individuals before the sampling. *Megasphaera* sp. isolates NM10 and BL7 are deposited with DSMZ (Deutsche Sammlung von Mikroorganismen und Zellkulturen, Germany) under accession numbers DSM25563 and DSM25562 respectively. The 16S rRNA gene phylogeny revealed that these strains belong to the family *Veillonellaceae* and showhigh similarity with *Megasphaera hominis* and *M. elsdenii*. However, *M. hominis* is not included in the list of validly published species (http://www.bacterio.net) and is not in the list of prokaryotic names with standing nomenclature. Therefore, we considered the closest validly published type strain, that is, *Megasphaera elsdenii* (DSM20460) (Figure 1). The 16S rRNA gene sequences of *Megasphaera* sp. NM10 and BL7 are deposited at GenBank under accession numbers HM990965 and HM990964 respectively. Recently, *Megasphaera massiliensis*, a novel species belonging to genus *Megasphaera* was proposed and its genome sequence has been described [13]. The 16S rRNA gene sequences of *Megasphaera* sp. NM10 and BL7 were 96% similar to 16S rRNA sequence of *M. massiliensis,* suggesting that these human gut isolates belong to a different species. Hence, the genome of *M. massiliensis* was not considered for comparative genome analysis. The polyphasic taxonomy suggested that the isolates *Megasphaera* sp. NM10 and BL7 represent a novel species belonging to genera *Megasphaera* (data not shown).

### General Features of *Megasphaera* sp. NM10 and *Megasphaera* sp. BL7 Genomes

The draft genomes of *Megasphaera* sp. NM10 and BL7 are deposited at GenBank under accession numbers APHY00000000 and APHX00000000 respectively. The draft genome of *Megasphaera* sp. NM10 was 2,615,280 bp and that of *Megasphaera* sp. BL7 was 2,656,480 bp with a G+C content of approximately 54.3% for both NM10 and BL7. The sequencing coverage obtained for the genomes of NM10 was 74X and for BL7 was 83X. The total consensus length of draft genomes obtained for both of the isolates in this study is larger than the genome sequence available for the type strain *M. elsdenii* DSM20460 (∼2.47 Mb). The functional annotation of sequence data was performed using RAST server that uses a subsystem-based approach [14]. The number of subsystems identified for NM10 were 297 and the predicted number of open reading frames (ORF’s) were 2436. For BL7, the number of subsystems identified were 293 and the predicted number of open reading frames (ORF’s) were 2432. In addition to these features, phage elements were detected in both the NM10 and BL7 genome sequence assembly. The publicly available genome of *M. elsdenii* DSM20460 (accession number HE576794) was also reanalyzed using RAST, for having consistency in comparison [11]. The subsystems features predicted by RAST server based on the genome sequences of NM10, BL7 and *M. elsdenii* DSM20460 are represented in the Table 1.

**Table 1:** The predicted sub-system features in the genomes of *Megasphaera* sp. NM10, BL7 and *M. elsdenii* DSM20460.

Subsystem Feature	Megasphaerasp. NM10	Megasphaerasp. BL7	M. elsdeniiDSM20460
Cofactors, Vitamins, Prosthetic Groups, Pigments	123	123	141
Cell Wall and Capsule	103	102	108
Virulence, Disease and Defense	44	49	45
Potassium metabolism	15	15	15
Phages, Prophages, Transposable elements, Plasmids	14	12	5
Membrane Transport	44	44	35
RNA Metabolism	110	107	115
Nucleosides and Nucleotides	71	107	75
Protein Metabolism	162	134	136
Cell Division and Cell Cycle	21	21	21
Regulation and Cell signaling	8	8	8
Secondary Metabolism	6	0	6
DNA Metabolism	84	79	94
Regulons	3	2	3
Fatty Acids, Lipids, and Isoprenoids	43	43	46
Nitrogen Metabolism	13	14	16
Dormancy and Sporulation	2	2	2
Respiration	46	46	47
Stress Response	44	44	43
Metabolism of Aromatic Compounds	4	4	4
Amino Acids and Derivatives	273	271	273
Phosphorus Metabolism	33	32	33
Carbohydrates	255	219	201
**Miscellaneous**			
Niacin-Choline transport and metabolism	4	4	3
Phosphoglycerate mutase protein family	3	3	2
Muconate lactonizing enzyme family	1	1	1

**Figure 2:**
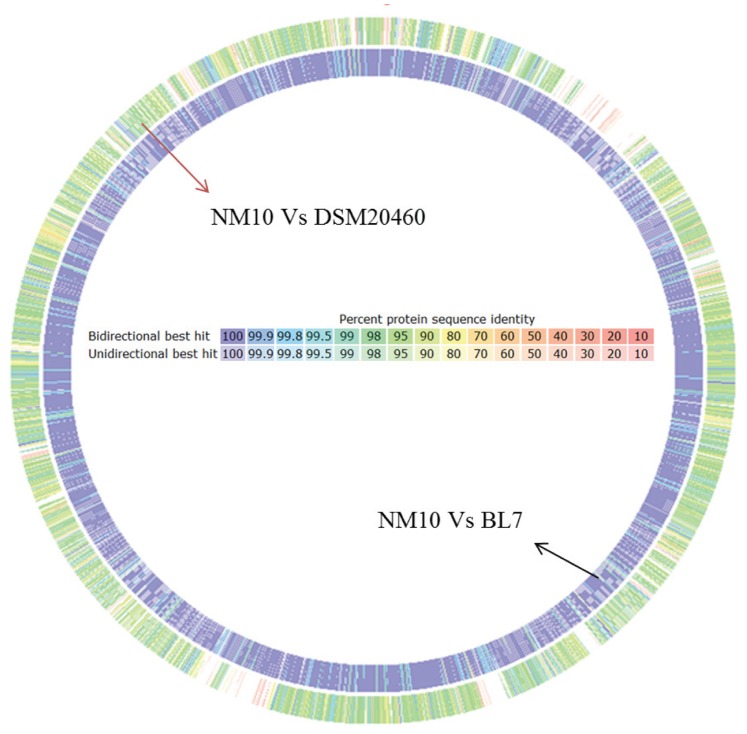
Comparison of protein sequences of *Megasphaera* sp. NM10, BL7 and *M. elsdenii* DSM20460. The color code indicates the percent similarity between the predicted protein sequences.

The comparison of protein sequences of all predicted ORF’s in the genomes of NM10, BL7 and *M. elsdenii* DSM20460 showed that the genomes of human gut isolates NM10 and BL7 are highly similar to each other, with around 2200 proteins sharing more than 99.5% similarity between them (Figure 2 and Table S1). Whereas *M. elsdenii* genome had low sequence similarity to the genomes of the human gut isolates, with only 252 proteins having more than 99% similarity to the human gut isolates, suggesting that the genomes of human gut isolates differ from ruminal isolate DSM20460. In addition, more than 400 proteins encoded by the genomes of the human gut isolates were not detected in the ruminal isolate (Table S1).

### General Metabolic Potential of *Megasphaera* sp. NM10 and BL7

The draft metabolic model for the three *Megasphaera* sp. obtained from Model SEED suggested the potential of these isolates to produce various primary and secondary metabolites. The predicted genes by RAST for NM10, BL7 and DSM20460 are enlisted in Table S1. The Model SEED predicts the presence of a pathway based on the presence of a protein or a single step in the pathway. The genomes under study were unclosed drafts; this would lead to imprecise estimation of the metabolic capabilities of these bacteria. To avoid this inflated estimate of the biological pathways and the metabolic capabilities of the bacteria, we carried out MinPath (Minimal set of Pathways) analysis. The MinPath analysis uses protein family predictions for biological pathway reconstructions; this yields a more conservative and more faithful, estimation of the biological pathways for a query dataset [15]. This assisted in avoiding overestimation of the functional capabilities of the isolates and to have a more conservative and concrete prediction of the metabolic capabilities based on draft genomes. The metabolic functions predicted for all three isolates by MinPath analysis are given in Tables S2, S3 and S4.

#### Central metabolism

The human gut metagenomic and metatranscriptomic studies have revealed that the genes for central metabolic pathways like carbohydrate metabolism, amino acid metabolism, nucleotide metabolism, etc., are abundantly expressed in the human microbiome [5], [16]–[18]. The general metabolic features of *Megasphaera* sp. NM10 and BL7 were highly similar to each other and most of the predicted features in the subsystem are shared by these isolates.

#### Carbohydrate metabolism

The genomes of NM10 and BL7 coded for enzymes essential for carrying out glycolysis and gluconeogenesis. Out of the total 42 SEED families for glycolysis and gluconeogenesis, 11 were present in NM10 and 10 were identified in BL7. The genomes also had genes coding for enzymes of Tricarboxylic acid cycle, Pentose phosphate pathway and Entner-Doudoroff pathway. Out of the 56 SEED families for the Entner-Doudoroff pathway, 14 were represented in NM10 and BL7. Both the isolates were capable of utilizing fructose and had genes coding for 1-phosphofructokinase (EC 2.7.1.56), a PTS system (EC 2.7.1.69), fructose-specific IIA component, fructose-specific IIB component, and fructose-specific IIC component. These isolates were also capable of mixed acid fermentation. Butyryl-CoA dehydrogenase (EC 1.3.99.2) was present in both the genomes; this enzyme is involved in the Acetyl-CoA fermentation to produce butyrate. NM10 and BL7 genomes had higher number of genes involved in carbohydrate metabolism as compared to the ruminal isolate DSM20460 (255 and 219 compared to 201) (Table 1), this may be attributed to the difference in host diet.

#### Amino acid and nucleotide metabolism

Amino acids are broadly classified as non-essential and essential amino acids**.** The latter group of amino acids are the ones that the human body cannot synthesize and consequently depends largely on food and the microbiome as its source [19]. The genomes of NM10 and BL7 had 273 and 271 subsystem counts associated with subsystem feature for amino acid metabolism. The MinPath analysis predicted the capability of these isolates for the biosynthesis of essential amino acids like histidine, lysine, methionine, threonine and tryptophan. The synthesis of lysine was predicted *via* the diaminopimelate **(**DAP) pathway which leads to the production of lysine from aspartate [20]. The important feature of this pathway is the intermediate diaminopimelate, which is important for peptidoglycan synthesis. In addition to the essential amino acids, the genomes of isolates NM10 and BL7 encode for the biosynthetic pathways for the synthesis of non-essential amino acids such as glycine (biosynthesis from L-threonine by threonine aldolase), arginine (*via* the arginine biosynthesis extended), glutamine, glutamate, aspartate, asparagine, cysteine (produced via super pathway of cysteine biosynthesis), proline (using glutamate as substrate to obtain proline) and homoserine. The Model Seed predicted the ability for synthesis of all three branched-chain amino acid (leucine, isoleucine and valine) but, the MinPath analysis suggested that these isolates were incapable for the same. The presence of membrane transporters for branched chain amino acids in the genomes of these isolates would aid in acquisition of these branched-chain amino acids.

In case of nucleotide synthesis, the genomes of the isolates NM10 and BL7 had genes involved in *De Novo* purine and pyrimidine synthesis pathways. The enzymes required to generate 5-phosphoribosyl-1-pyrophosphate i.e. ADP-ribose pyrophosphatase (EC 3.6.1.13), Ribose-phosphate pyrophosphokinase (EC 2.7.6.1) were present in the genomes of NM10 and BL7.

### Adaptive Features of *Megasphaera* sp. NM10 and BL7 for Survival in Human Gut

Mammalian gut is one of the most densely populated ecosystems in which the microbial populations are governed by a dynamic process of selection and competition [21], [22]. Survival in such a challenging ecosystem necessitates the bacteria to constantly adapt and evolve. In the human gut these different adaptive features include presence of carbohydrate degrading genes, resistance to stress conditions, sensing the surroundings, membrane transporters, etc.

#### ‘Glycobiome’

The entire gene repertoire involved in the breakdown of carbohydrates is termed as the ‘glycobiome’ [23]. Studies on the human gut metagenome has revealed the presence of an extensive glycobiome harbored by the gut microbial community [24], [25]. This feature has been studied in most of the bacteria associated with the human gut. The most studied bacterium with the largest reported glycobiome is *Bacteroides thetaiotaomicron.* This bacterium encodes more than 170 glycosylhydrolases [22]. Similarly, other gut symbionts add up to the glycobiome potential of the gut microbiome.

**Figure 3:**
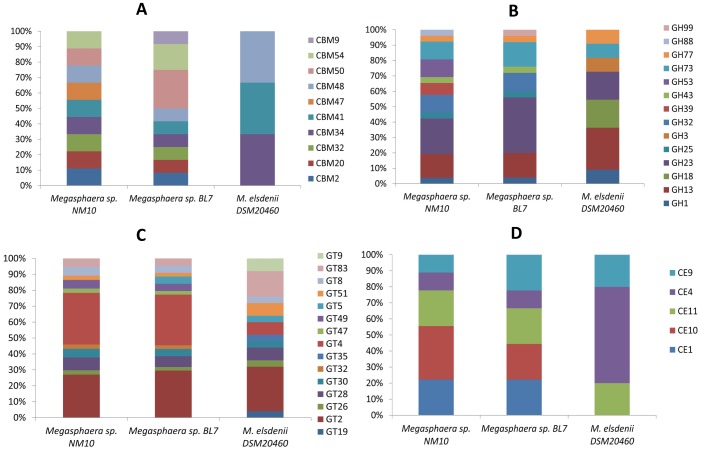
The distribution of different CAZyme families in genome of *Megasphaera* sp. NM10, BL7 and *M. elsdenii* (DSM20460). A) Distribution of Carbohydrate-Binding Modules (CBMs). B) Distribution of Glycoside Hydrolases (GHs). C) Distribution of Glycosyl Transferases (GTs), D) Distribution of Carbohydrate Esterases (CEs).

Comparisons of the glycobiome of the *Megasphaera* sp. NM10 and BL7 of the human fecal origin with that of *M. elsdenii* DSM20460 of the ruminal origin, showed the presence of a higher repertoire of complex carbohydrate utilizing genes in the human gut isolates (Figure 3). The Polysaccharide Lyase Family (PLs) was not detected in all the three *Megasphaera* sp. genomes. *M. elsdenii* is not a primary metabolizer in the rumen, but rather is involved in utilizing the end products of fermentation especially; lactic acid [26]. *Megasphaera* sp. might play a similar role in the human gut.

**Figure 4:**
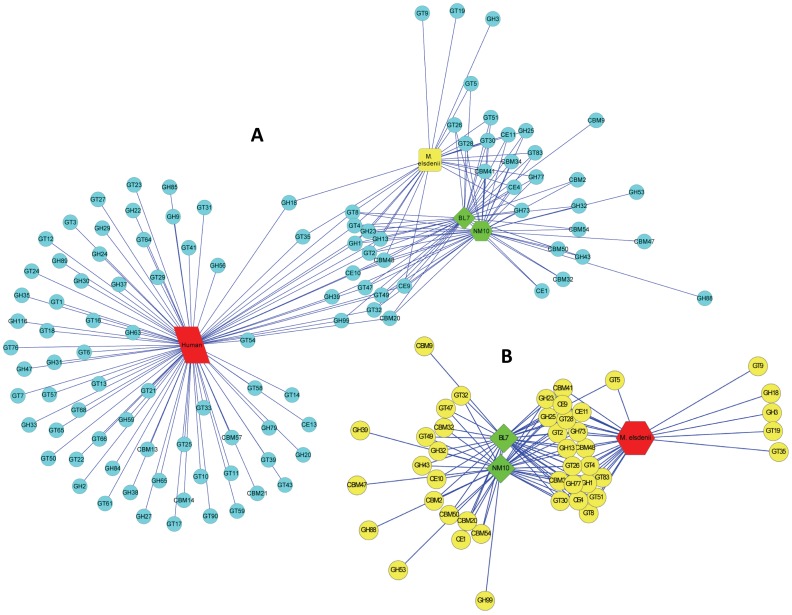
The Glycobiome network of *Megasphaera* sp. A) The glycobiome network of Human (red), *Megasphaera* sp. NM10, BL7 (green) and *M. elsdenii* DSM20460 (yellow). B) The glycobiome network of *Megasphaera* sp. NM10, BL7 (green) and *M. elsdenii* DSM20460 (red). CBMs- Carbohydrate-Binding Modules, GHs- Glycoside Hydrolases, GTs- Glycosyl Transferases, CEs- Carbohydrate Esterases. The nodes represent the CAZyme superfamilies and the edges are connecting the nodes based on the presence or absence of respective superfamilies in the organism.

The Carbohydrate-Active EnZymes (CAZymes) of *Megasphaera* sp. NM10 and BL7 were compared to the CAZymes that are encoded by the human genome (Figure 4A). The human genome encodes 29 glycosyl hydrolases families. However, only a few of these enzymes are involved in the digestion of carbohydrates present in the daily diet. On comparing the glycobiome of the human genome and the ruminal isolate with the human gut isolates, it was observed that the human gut isolates encode enzymes that help fill the enzymatic lacunae required to degrade carbohydrates present in the human diet. These enzymes belong the glycosyl hydrolase (GH) families GH25, GH32, GH43, GH53, GH73 and GH77 (GH88 was detected only in NM10) thereby adding to the potential of the gut microbiome for degrading various carbohydrates. This observation suggests that *Megasphaera* sp. NM10 and BL7 play a role in utilization of carbohydrates that the host is incapable of degrading. A study by Cantarel *et al* in 2012, suggested that the gastrointestinal tract has the highest abundance of CAZymes among all the body sites [24]. The CAZy family GH53 (involved in plant cell wall degradation), is among the six over-represented families in the digestive tract. GH53 was present in the genome of isolate NM10. The human gut isolates also carried the genes that encode amino acid sequence having carbohydrate-binding activity. These are classified as carbohydrate-binding module (CBM). The genomes of NM10 and BL7 encoded glycosyl hydrolase GH13 associated with CBM48 and CBM41, which help in binding to starch. In addition, the *Megasphaera* sp. NM10 and BL7 genomes also encoded amylomaltase that is associated with CBM34, which is a starch binding domain.

The glycobiome network (represented in Figure 4B) indicated that only a few CAZymes are shared between the rumen and the human gut isolates. The ruminal isolate genome encoded glycosyl hydrolases, GH18 (a conserved domain protein) and GH3 (beta-glucosidase-related glycosidases), glycosyltransferases GT19 (lipid-A-disaccharide synthase), GT9 (lipopolysaccharide heptosyltransferase I) and GT35 (phosphorylase). However, eighteen CAZyme families were observed to be present only in the human gut isolates. These included various glycosyl hydrolases such as GH43, GH32, GH99, GH53, GH88, and GH39 and glycosyltransferases like GT32, GT47 and GT49. This could be attributed to adaptation to the host specific diet. The human diet is diverse as compared to the ruminals. Diet of humans contains different sources of carbohydrates ranging from simple sugars to complex polysaccharides. Consequently, to adapt to these diverse sources of carbohydrates the bacteria in the human gut need to have a higher repertoire of genes involved in carbohydrate degradation. The unique set of CAZymes present in human gut isolates and the diverse CAZyme repertoire compared to ruminal isolate signify a probable host specific evolution and adaptation of the genomes of the isolates of *Megasphaera* sp. isolated from the human gut.

#### Bile resistance

One of the selective pressures in the gut is the presence of bile. The bacteria that are sensitive to bile are not capable of survival in the gut. The bile tolerance test suggested that all the three isolates in this study can tolerate 0.3% bile. Bile salt hydrolase (*bsh*) is the enzyme responsible for bile resistance in many bacteria [27]. The bile salt hydrolase activity was not detected in the human gut isolates as well as *M. elsdenii* in the *in vitro* assay, Figure S1. On analysis of the genome of all the three *Megasphaera* sp., the gene encoding bile salt hydrolase (*bsh*) was not detected [27]. This is consistent with the absence of bile salt hydrolase activity, in the *in vitro* analysis, among these isolates. However, bile acid: sodium symporter and bile acid transporters were identified in the genomes of both the human gut isolates. Additionally, several MDR pumps conferring bile resistance along with resistance to drugs were detected in the genomes of NM10 and BL7. These include MDR efflux pump *CmeABC* which is essential for bile resistance in *Campylobacter* [28], [29]. The *acrAB* has been reported to confer bile resistance in *Salmonella typhi* [30], while *TolC* efflux pump is reported to confer bile resistance in *Vibrio cholerae* [31]. Presence of all these efflux pumps, explains the observed bile resistance detected in these isolates.

#### Oxidative stress

Presence of oxygen and reactive oxygen species is a major stress for obligate anaerobic organisms. The genomes of the isolates NM10 and BL7 had several mechanisms for protection against oxidative stress. These include the presence of glutathione peroxidase and glutathione-dependent enzyme systems like lactoylglutathione lyase (EC 4.4.1.5) and hydroxyacylglutathione hydrolase (EC 3.1.2.6) (also called glyoxalase I and glyoxalase II), these enzymes detoxify oxidative stress and are important for bacterial survival [32], [33]. The Ferritin-like Dps protein, transcriptional regulator Rex and peroxide stress regulator PerR belonging to FUR family are known to regulate oxidative stress response in bacteria, all these genes were detected in the genomes of the isolates NM10 and BL7 [34]–[36]. Rubrerythrin was found to be one of the abundant proteins in human gut metaproteomic studies [5]. Rubrerythrin and superoxide reductase (EC 1.15.1.2) system are shown to be involved in the protection against oxidative stress [37]. These systems were present in the genomes of the isolates NM10 and BL7. Thus, the isolates NM10 and BL7 have various oxidative stress management systems that aid in the survival of these isolates in the gut environment.

#### Stress due to antibiotics

In addition to the internal selective pressures, external factors such as antibiotics pose a major challenge for the survival of bacteria in the human gut. This is mainly because of the constant selective pressure due to consumption of antibiotics during the treatment of infections. Studies have demonstrated that the human gut microbiota can act as a reservoir of antibiotic resistance [38], [39]. *Megasphaera* sp. NM10 and BL7 harbor multidrug resistance efflux pumps and genes that confer resistance to specific antibiotics. The genome of NM10 encodes for genes that confer resistance to beta-lactams, quinolones, fosmidomycin, polymyxins, macrolides and vancomycin. BL7 has genes for resistance to vancomycin, quinolones, macrolides and metallo-betalactamases. These genes would give adaptive advantage for these isolates during antibiotic treatment of the host.

Horizontal gene transfer (HGT) is considered as one of the major factors contributing to increased antibiotic resistance in the human gut commensals. Antibiotic resistance genes are associated with mobile elements such as plasmids and transposons, and are frequently transferred between gut commensals [40], [41]. The presence of plasmid conjugal transfer proteins, mobile element proteins and transposes in the genomes of isolates NM10 and BL7 suggest that these genes are acquired by human gut isolates for adaptation and survival in human gut. All these genes were not detected in the genome of *M. elsdenii* DSM20460 (Table S1).

#### Sensing the surroundings

Sensing the environmental metabolites is an important factor in the survival of the bacteria in any environment, as it is associated with various responses such as uptake of the available nutrients [42]. Both NM10 and BL7 genomes had an elaborate repertoire of genes encoding proteins associated with sensory response and transcriptional regulation. Sigma factors viz. sigma factor –54 and sigma factor –70, that are involved in sensing the environmental clues, were detected in both the isolates [42]. In addition to sigma factors, genes encoding substrate specific sensory proteins were identified in the genomes of *Megasphaera* sp. NM10 and BL7. Various two-component systems were identified in the genomes of NM10 and BL7. The two-component regulatory systems play a crucial role as regulators of various environmental signaling transduction pathways. These two-component systems have a histidine kinase sensor containing histidine kinase and phosphoacceptor domains that is localized in the membrane [43]. PhoR-PhoB two-component regulatory system associated with a high affinity phosphate transporter was detected in the *Megasphaera* genomes. Both NM10 and BL7 had a two-component sensor regulator linked to the carbon starvation protein-A, belonging to sensory box/GGDEF family protein and several histidine kinases. A hybrid two component regulatory system which is an AraC (arabinose specific helix-turn-helix domain) i.e. arabinose operon control protein; was present in both NM10 and BL7. This hybrid two component system with DNA binding domain was first identified in *B*. *thetaiotaomicron,* which consisted of 32 of these novel hybrid histidine kinases with a DNA-binding domain [22]. The hybrid two-component regulatory system present in the isolates NM10 and BL7 is for rhamnose utilization, highlighting the fact that these isolates have an ability to sense the presence of a carbon and energy source in the gut environment. In addition, the genomes of NM10 and BL7 have a methyl-accepting chemotaxis protein. Methyl-accepting chemotaxis proteins are a class of sensory receptors mediating chemotaxis to diverse extracellular and intracellular signals [44].

The presence of various sensory and regulatory systems suggests that the isolates NM10 and BL7 have the features required to sense the available nutrients in the environment and subsequently regulate a wide array of genes for the uptake and utilization of these nutrients. This would help *Megasphaera* sp. to overcome one of the major challenges in the complex gut environment i.e., the ability to distinguish between varied environmental chemical molecules.

#### Membrane transporters

Membrane transporters are important for the survival of bacteria in any environment. These membrane transporters facilitate the exchange of nutrients and metabolites with the surrounding environment. ATP-binding cassette transporters (ABC-transporter) are the most prevalent membrane transporters found in all organisms, involved in the transport of various substrates across cell membrane [45]. ABC-transporters for several substrates were detected in the genomes of isolates NM10 and BL7, these included transporters for important substrates like sugars, phosphate, iron, zinc, sulphate, nickel, molybdenum and important metabolites like amino acids, vitamin B12, spermidine-putricine and formate/nitrate (Table S1). The presence of D-serine/D-alanine/glycine, glutamine ABC transporter, methionine ABC transporter permease protein, serine transporter, transporters for aromatic, branched chain amino acid and several other amino acid transporters was detected in both the isolates. The MinPath analysis predicted the inability of the human gut isolates to synthesize serine and alanine. The presence of transporters for serine and alanine would facilitate acquisition of serine and alanine by these isolates from the environment and thereby help in the survival of these isolates.

Oligopeptide transport systems are important systems that facilitate uptake of oligopeptides from the environment. The presence of opp operon (Opp*ABCDF*) for oligopeptide transporter, suggests the ability of NM10 and BL7 to compete in the gut environment with an ability to use various sources of amino acids.

Another family of transporters, the major facilitator superfamily (MFS) involved in solute transport, was detected in both the isolates [46]. These include nitrate/nitrite transporter, Na+/H+ antiporter NhaA type, formate efflux pump and sugar efflux pump. Drug/metabolite efflux pumps of MATE family and RND type were detected, these efflux pumps along with magnesium and cobalt efflux protein CorC and Co/Zn/Cd efflux system may be responsible for protection against antibiotics and heavy metals.

#### Phage associated genes

**Figure 5:**
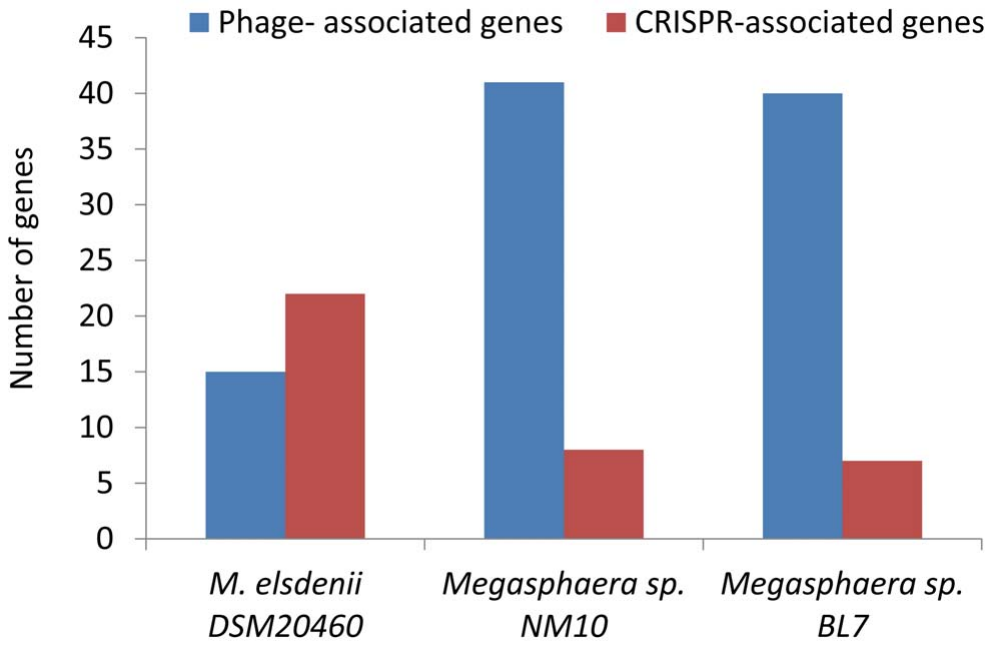
Distribution of CRISPRs and phage-associated genes in the genomes of *Megasphaera* sp. NM10, BL7 and *M. elsdenii* DSM20460.

Along with the bacterial population, the human gut harbors viruses. In order to survive in such ecosystem, bacteria have evolved defense mechanisms that help them in counter acting the infection by viruses [47]. Two of these mechanisms involve prokaryotic restriction–modification (R/M) systems and clustered regularly interspaced short palindromic repeats (CRISPRs), which give protection against foreign DNA like phages and plasmids [48], [49]. CRISPRs are short, direct repeating sequences (typically 30–40 nucleotide long) that separate variable sequences of similar size. These act as a database of fragments derived from phage and plasmid genomes and provide protection to the bacteria [49]. The genomic analysis revealed the presence of different CRISPR families in all the three studied *Megasphaera* sp. genomes. The highest number was detected in the ruminal isolate; on the contrary relatively fewer CRISPRs were detected in the human gut isolates (Figure 5 and Table S1). Consequently, the number of phage-associated genes in the ruminal isolate was comparatively lower to the human gut isolates, suggesting a higher resilience to phage attack in ruminal isolate. Metagenomic study of the human gut microbiome showed that approximately 5% of the genes in the gut metagenome were phage associated genes [16]. This study speculated a probable role of pro-phages in the evolution of gut microbiome. In order to adapt to the human gut environment, carrying mobile DNA such as plasmids (invariably carrying antibiotic resistance genes) and phages seem to be important for adaptation to stress. The presence of the CRISPR elements in the human gut isolates suggests the ability of these bacteria to sustain attack from specific bacteriophages, while allowing exchange of some mobile DNA; this is an important adaptation for survival in the gut ecosystem. The human gut isolates harbor some type II and III restriction modification systems, which were absent in the genome of ruminal isolate (Table S1), suggesting presence of an alternative mechanism for the defense against phages.

### Potential Beneficial Effects on the Host

The beneficial effect of the gut microbes on the host is by and large through the metabolites utilized and/or produced through various metabolic pathways. These range from fermentation end products (mostly short chain fatty acids), vitamins and co-factors to numerous other bio-molecules [50]. In the present study, we identified the metabolic capabilities as defined by the genomic data for production of such important metabolites and validated by detecting the *in vitro* production.

#### Short Chain Fatty Acids (SCFA’s) production

**Figure 6:**
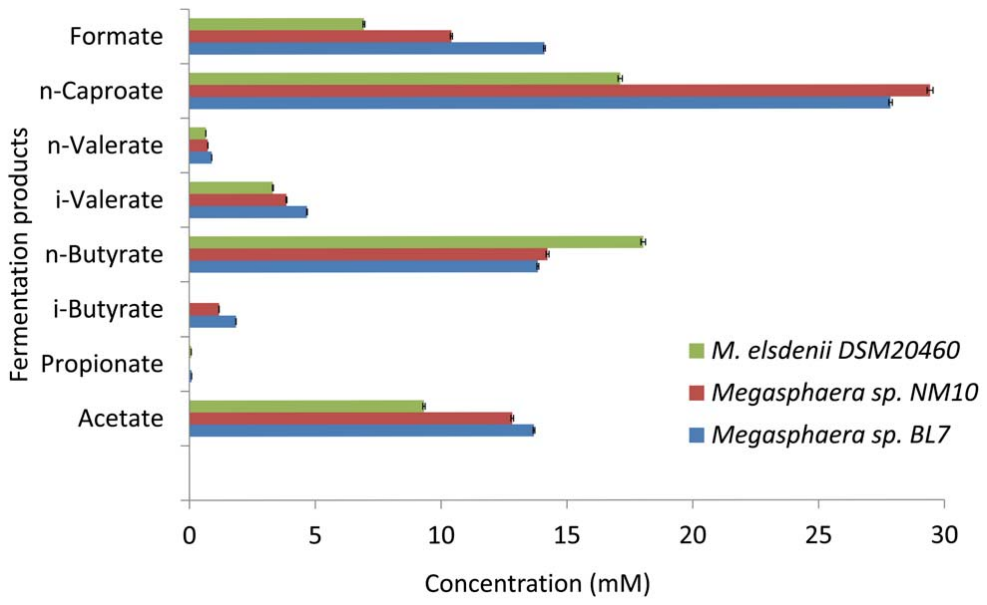
Fermentation products of glucose utilization by *Megasphaera* sp. NM10, BL7 and *M. elsdenii* DSM20460. The error bar represents standard deviation of three technical repliactes.

**Figure 7:**
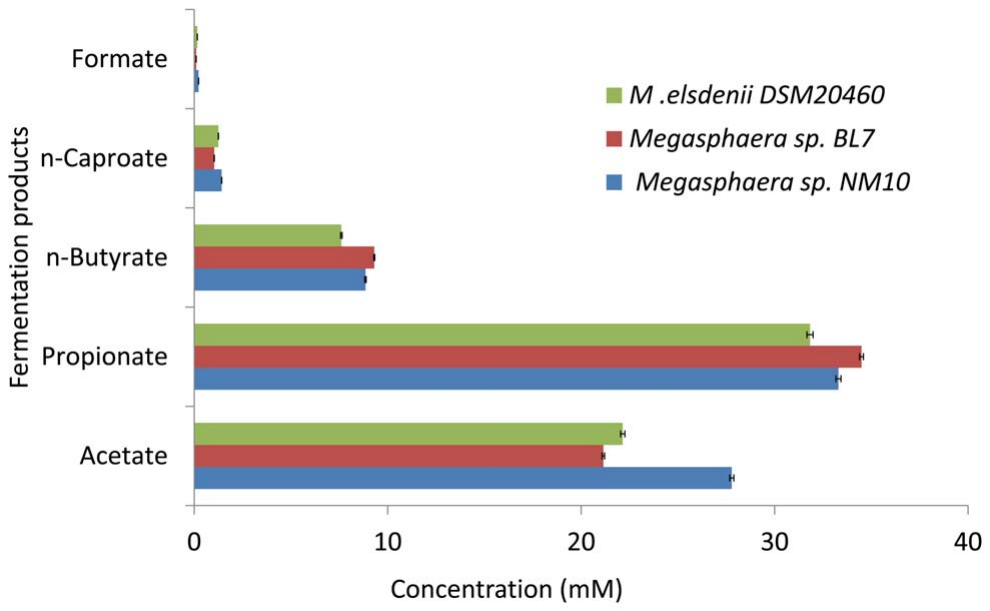
Fermentation products of lactate utilization by *Megasphaera* sp. NM10, BL7 and *M. elsdenii* DSM20460. The error bar represents standard deviation of three technical repliactes.

The SCFA’s are products of degradation of the dietary fibers by bacteria. Most prominent SCFA’s produced in human gut are acetate, butyrate, propionate and valerate [50]. The *In silico* metabolic analysis predicted the ability of the human gut isolates NM10 and BL7 to synthesize SCFA’s like butyrate, formate, acetate and valerate. Analysis of the fermentation products of these bacteria confirmed the ability of these isolates to produce acetate, butyrate, valerate and formate (Figure 6 and 7). The glucose fermentation yielded butyrate, formate, acetate, valerate and caproate; while negligible amounts of propionate were produced (Figure 6). Caproate was the major product of glucose fermentation. Human gut isolates produced ∼3.5 mg/L caproate, which is significantly higher than ruminal isolate (∼1.98 mg/L). Caproate is an industrially important product and serves as a precursor for cholesterol synthesis and synthesis of hormone progesterone [51].

Lactate is produced by microbes colonizing human gut as the end product of carbohydrate fermentation. Lactate accumulation is observed in short bowel syndrome and ulcerative colitis, this accumulation can be serious, causing neurotoxicity and cardiac arythmia [52], [53]. A study in porcine cecal digesta has shown that a co-culture of lactic acid producing bacteria, *Lactobacillus acidophilus* and *M. elsdenii* stimulates butyrate production [54]. *M. elsdenii* is known to produce SCFA’s by utilizing lactate and reduce acidosis in ruminals [55], [56]. Utilization of lactate by *Megasphaera* sp. may serve a similar function (reducing lactate toxicity and producing important metabolites like SCFA’s) in human gut. In order to validate this, SCFA’s production by NM10, BL7 and *M. elsdenii* by utilizing lactate was checked. In contrast to glucose fermentation, propionate was the major product of lactate fermentation followed by acetate and butyrate (Figure 7). Negligible amount of formate was detected on lactate utilization, while low amount of caproate was produced.

Many of these SCFA’s have a positive effect on the human health. SCFA’s stimulate water and sodium absorption in the epithelial cells. SCFA’s play an important role in the proliferation, differentiation and regulation of gene expression in the epithelial cells. Butyrate and acetate are used as an energy source by the colonic epithelium and the muscles [50], [57], [58]. In addition, acetate, propionate and to some extent butyrate, act as ligands for signaling molecules. Propionate and acetate are transported to liver through blood stream and used for gluconeogenesis [50]. The production of these SCFA’s by NM10 and BL7 suggests that these isolates may have a potential positive effect on the host health. Caproate has a protective effect on the host, it is reported to reduce colonization of pathogens in the gut [59]. Formate and caproate production would give competitive advantage to these isolates in human gut as formate and caproate are reported to have antibacterial activity against both Gram-negative and Gram-positive organisms [60].

**Figure 8:**
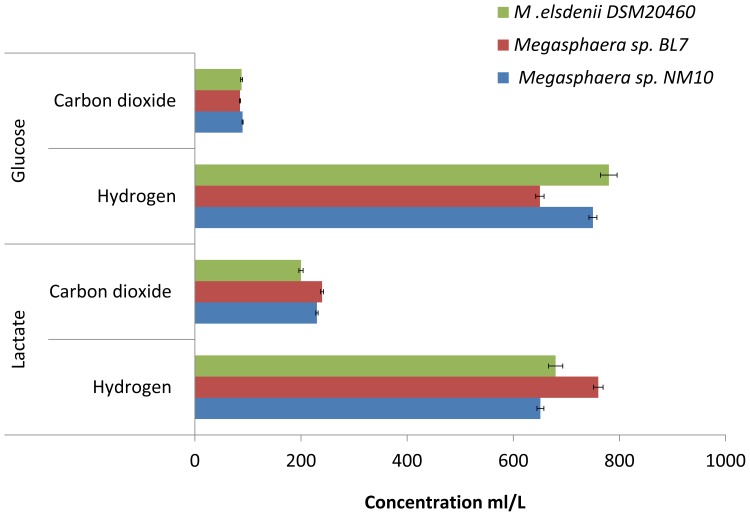
Gas production by *Megasphaera* sp. NM10, BL7 and *M. elsdenii* DSM20460 using glucose and lactate as substrates. The error bar represents standard deviation of three technical repliactes.

Hydrogen and carbon dioxide are produced as a byproduct of fermentation by both NM10 and BL7 using both glucose and lactate as substrates (Figure 8). Hydrogen and carbon dioxide produced during fermentation are utilized by methanogens, while hydrogen is utilized by acetogens and sulphate-reducing bacteria as a substrate for metabolism [61]–[63]. This makes *Megasphaera* sp. an important part of the food chain in the human gut environment.

#### Production of vitamins

The microbes serve as a complementary source of some vitamins and a primary source for other vitamins such as biotin, pyridoxine and its derivatives which, the humans are not capable of synthesizing [64]. RAST annotation of the genomes identified genes coding enzymes for production of various vitamins such as biotin, thiamine, folate, pyridoxine, niacin, riboflavin. The MinPath analysis assigned functional annotations for biosynthesis of five different vitamins *viz*. biotin, thiamine, folate, B12 and pyridoxine. In case of riboflavin, the gene encoding riboflavin synthase was detected. The *ribAH* operon for riboflavin synthesis was present in both the isolates. This suggests that these isolates are capable of converting riboflavin into flavin mononucleotide *via* riboflavin kinase and flavin mononucleotide to riboflavin via a putative phosphotyrosine protein phosphatase. Entire pathway for vitamin B12 production was identified in the genomes of the isolates NM10 and BL7. The production of vitamin B12 by these isolates was validated by the *in vitro* assay. The isolate BL7 produced 3.83 ng vitamin B12/g of cell mass, while NM10 produced 6.52 ng vitamin B12/g of cell mass respectively. Thus, *Megasphaera* sp. can potentially provide vitamins to the host.

## Conclusion

In the present study, we sequenced genomes of two potential novel *Megasphaera* sp. (NM10 and BL7) that were isolated from stools of two healthy Indian individuals. Previously, we have discussed the importance of gut microbiome studies in the Indian population [65]. The significance of developing population specific indigenous probiotics by exploring novel bacteria from the human gut has also been highlighted. Comparative genome analysis of two potential novel isolates of the *Megasphaera* sp. (NM10 and BL7) and *Megasphaera elsdenii* (DSM20460), in the current study, demonstrates the differences in genomic capabilities of these bacteria. Previous study on the genomes of *Bacteroides* species suggested that the variation between genomes may help the bacterium to evolve for a specific niche in the gut environment [22]. The genomic differences observed in this study may be attributed to the niche specialization and adaptation of the human gut isolates for survival in the gut environment. The observed difference between the genomes of the human gut isolates and the ruminal isolate, and similarities amongst the human gut isolates; demonstrates the evolutionary delineation of genomes towards adaptation to the human gut ecosystem. The human gut isolates are characterized by the presence of an enriched set of CAZymes compared to their ruminal counterpart. Additionally, the presence of various stress related genes, sensory systems, membrane transporters and resistance to antimicrobials provide an evidence for their adaptation in order to survive in the human gut and interact with the intestinal microbial community. The metabolic features of the human gut isolates suggest that these isolates play an important role in the complex gut environment, and in part, do add to the overall metabolic functions of the human gut microbiome. The human gut isolates have an ability to produce essential amino acids, vitamins and utilize lactic acid to produce SCFA’s. Overall, the study highlights the crucial adaptive features of *Megasphaera* sp. NM10 and BL7 for survival in the human gut and their potential for having a positive effect on the host health. The knowledge about the genome sequence of the two potential novel bacteria in this study is an addition to existing knowledge about the metabolic and genomic capabilities of the bacterial species from the human gut. This would help in further understanding the role of different bacterial species found in the human gut environment. This study also provides a basis for future *in vivo* studies, using these isolates for better understanding the host-microbe interaction and confirming the predicted beneficial effect of these isolates on the host.

## Materials and Methods

### Genomic DNA Extraction, 16S rRNA Sequencing and Genome Sequencing

The cultures were grown in Peptone Yeast Glucose (PYG) medium under anaerobic conditions at 37°C. The DNA was extracted from freshly grown cultures using standard Phenol:Chloroform method [66]. Additionally, RNase treatment was given to obtain RNA free DNA from these isolates. The 16S rRNA gene sequencing was carried out as described earlier [12]. Phylogenetic analyses was carried out using MEGA, version 4 [67], and the phylogenetic tree was constructed using neighbor-joining method with Kimura 2 parameter with 1000 replicates [68]. For whole shot-gun genome sequencing (WGS) the purified genomic DNA was quantified using nanodrop ND-1000 spectrophotometer (JH Bio innovations, Hyderabad India). One µg of pure genomic DNA was fragmented into approximately 250 to 300 bp fragments. End-repaired fragmented DNA was used for adaptor ligation. Ligated DNA fragments were Size selected using E-Gel® SizeSelect™ 2% Agarose Gel (Invitrogen, USA) to prepare gDNA library. Size selected library was quantified using Agilent 2100 Bioanalyzer high-sensitivity chip (Agilent Technologies, Germany). The template dilution factor (TDF) was calculated and an ePCR with 26 pM DNA was done. The ISPs (Ion Sphere Particles) were purified and enriched before sequencing. Two 316 chips (one for each genome) were used for sequencing on Ion torrent PGM™ following the manufacturer’s protocol for 200 bp chemistry.

#### Assembly and annotation

The reads obtained in FASTAQ format were quality checked and assembled *de novo* using Mira assembler v3.0, this program relies on the overlap-layout-consensus approach, where each read is represented as a node and each detected overlap as an arch between the appropriate nodes [69]. Gene prediction and annotation were done using the RAST [70] server and the NCBI Prokaryotic Genomes Automatic Annotation Pipeline (PGAAP) (http://www.ncbi.nlm.nih.gov/genomes/static/Pipeline.html). RAST server is a rapidly growing, manually curated library of subsystems and annotation is based on protein families derived from the subsystems (FIGfams), RAST uses tRNAscan-SE for predicting tRNA genes [14], [71], [72]. The MultiGenomeCompare tool of the SEED was used for genome comparison where *Megasphaera sp* NM10 was used as the reference and was compared with BL7 and *M. eldesnii* DSM20460 [14]. This system computes a bidirectional BLAST comparison of each genome to the reference genome. The hits are given as bi- or uni- (bi stands for *bidirectional best hit* and uni for uni-directional.

#### Investigating biochemical potential and network based representation of glycobiome

The draft metabolic model was constructed using Model SEED version 1.0, which is a web-based resource for high-throughput generation, optimization and analysis of genome-scale metabolic models (theSEED.org). In addition, for a more faithful representation of metabolic capabilities of bacteria under study, MinPath (Bayesian method) analysis for prediction of metabolic capabilities was performed [15]. The glycobiome for *Megasphaera eldesnii* DSM20460 and Human was obtained from CAZy database (http://www.cazy.org/). Whereas, glycobiome of *Megasphaera* sp NM10 and *Megasphaera sp* BL7 was identified using CAZymes Analysis Toolkit (CAT) web-based server [73]. The comparative network of the glycobiome was constructed using Cytoscape v2.8 [74].

### *In vitro* Validation

#### Analysis of fermentation products

The isolates were grown in Peptone Yeast Glucose (PYG) and Peptone Yeast Lactic acid (PYL) broth for analysis of metabolic end products of glucose and lactate fermentation respectively. The metabolic end products were prepared as described by Holdeman *et al*. [75]. Short-chain fatty acids produced by the strains were extracted from 48 hrs broth cultures as previously described [76]. Volatile fatty acids were analyzed by Chemito 8610 GC equipped with Flame Ionization Detector, with oven temperature 150°C, injector temperature 170°C, detector temperature 190°C), column used was Chromosorb W (HP) (1.83 m×3.2 mm. SS) packed with 10% FFAP and 2% H3PO4. The carrier gas used was N2 at the flow rate of 30 ml min [77].

Gas production was determined in Peptone Yeast extract (PY), PYL and PYG broth using GC equipped with TCD [77]. H_2_ and CO_2_ were analyzed by Perkin Elmer GC (oven temperature 40°C, injector temperature 70°C, detector temperature 100°C ) using Porapak Q column, carrier gas used was Argon (Ar) at the flow rate of 40 ml min^−1^. In all the GC analyses, data analysis was done using IRIS 32 and total chrome navigations software. All experiments were carried out in triplicate.

#### Tolerance to bile

Brain Heart infusion yeast extract (BHI-YE) broth containing (per litre): 10 g BHI (Oxoid Ltd., England) supplemented with 10 g yeast extract, 10 ml hemin (Sigma-Aldrich, USA) solution (0.1%), 0.5 g cysteine HCl was used as the growth medium with pH 7 and N_2_ gas in headspace. Oxgall was added to this medium to get 3 concentrations of bile 0.2%, 0.3%, 0.4% (w/v), respectively. The BHI-YE broth was inoculated with 10% (v/v) of 48 hrs old culture and incubated at 37°C for 72 hrs. Bacterial growth was monitored every 24 hrs interval by measuring absorbance at 600 nm. Tolerance level of the strains was evaluated in terms of time required for increase in absorbance by 0.3 units (U) with respect to growth of the strains in the broth with and without oxgall [78].

#### Bile Salt Hydrolase (BSH) activity

BSH activity of the cultures was evaluated using the procedure described earlier [79]. Forty eight hrs old cultures were spot inoculated on BHI-YE medium plates supplemented with (per litre): 0.3 g oxgall (Sigma-Aldrich, USA) and 0.37 g CaCl_2_, 0.5 g cysteine HCl, 20 g Bacto Agar (Difco, USA). The diameters of precipitation zones were measured after incubation at 37°C for 72 hrs. BHI-YE agar plates without supplementation were used as controls. The strains which displayed the precipitation zone were considered positive for BSH activity.

#### Screening for vitamin B12 production

The fermentation was carried out in 100 ml Vitamin B12 production medium under anaerobic conditions at 37°C for 5 days [80]. Cyanide method was used for vitamin B12 extraction from bacterial cells [81]. The analysis of cell extract samples was conducted using HPLC system equipped with RP-C18 column 250×4.6 mm, 5 µm (LiChroCART, Germany) with an UV detector at 340 nm, 75% of 0.25 M NaH_2_PO_4_, pH 3.5, and 25% of methanol was used as the mobile phase with a flow of 1 ml min^−1^ at 40°C. Cyanocobalamin (Sigma, USA) was used as standard.

## Supporting Information

Figure S1 **The plate representing absence of bile salt hydrolase (BSH) activity in the isolates** ***Megasphaera*** **sp. NM10, BL7 and** ***M. elsdenii.*** The white zone of precipitation represents a positive result. A) Represents the absence of BSH activity in isolate NM10 and BL7, while presence of activity in the other isolates from the study. A) Represents the absence of BSH activity in isolate NM10, BL7 and *M. elsdenii*, *E. coli* is used a negative control.
(TIF)

Table S1 **Comparison and functions of all predicted protein sequences in** ***Megasphaera*** **sp. NM10, BL7 and** ***M. elsdenii*** **DSM20460 using MultiGenomeCompare tool.**
(XLS)

Table S2 **MinPath analysis results for** ***Megasphaera*** **sp. NM10.** Legend: The MinPath results are interpreted as follows. Naive 1 or 0: the pathway is reconstructed, or not, by the naive mapping approach; MinPath 1 or 0: 1-the pathway is kept, 0- removed by MinPath; fam0: the total number of families involved in the corresponding pathway; fam-found: the total number of involved families that are annotated; name: the description of the corresponding subsystem.
(XLS)

Table S3 **MinPath analysis results for** ***Megasphaera*** **sp. BL7.** Legend: The MinPath results are interpreted as follows. Naive 1 or 0: the pathway is reconstructed, or not, by the naive mapping approach; MinPath 1 or 0: 1-the pathway is kept, 0- removed by MinPath; fam0: the total number of families involved in the corresponding pathway; fam-found: the total number of involved families that are annotated; name: the description of the corresponding subsystem.
(XLS)

Table S4 **MinPath analysis results for** ***Megasphaera elsdenii*** **DSM20460.** Legend: The MinPath results are interpreted as follows. Naive 1 or 0: the pathway is reconstructed, or not, by the naive mapping approach; MinPath 1 or 0: 1-the pathway is kept, 0- removed by MinPath; fam0: the total number of families involved in the corresponding pathway; fam-found: the total number of involved families that are annotated; name: the description of the corresponding subsystem.
(XLS)
