# Supplementary figures and images for: Comparative Genome Analysis of Megasphaera sp. Reveals Niche Specialization and Its Potential Role in the Human Gut
Source: PLoS One. 2013 Nov 18;8(11):e79353. doi: 10.1371/journal.pone.0079353 (PMC3832451; doi:10.1371/journal.pone.0079353)

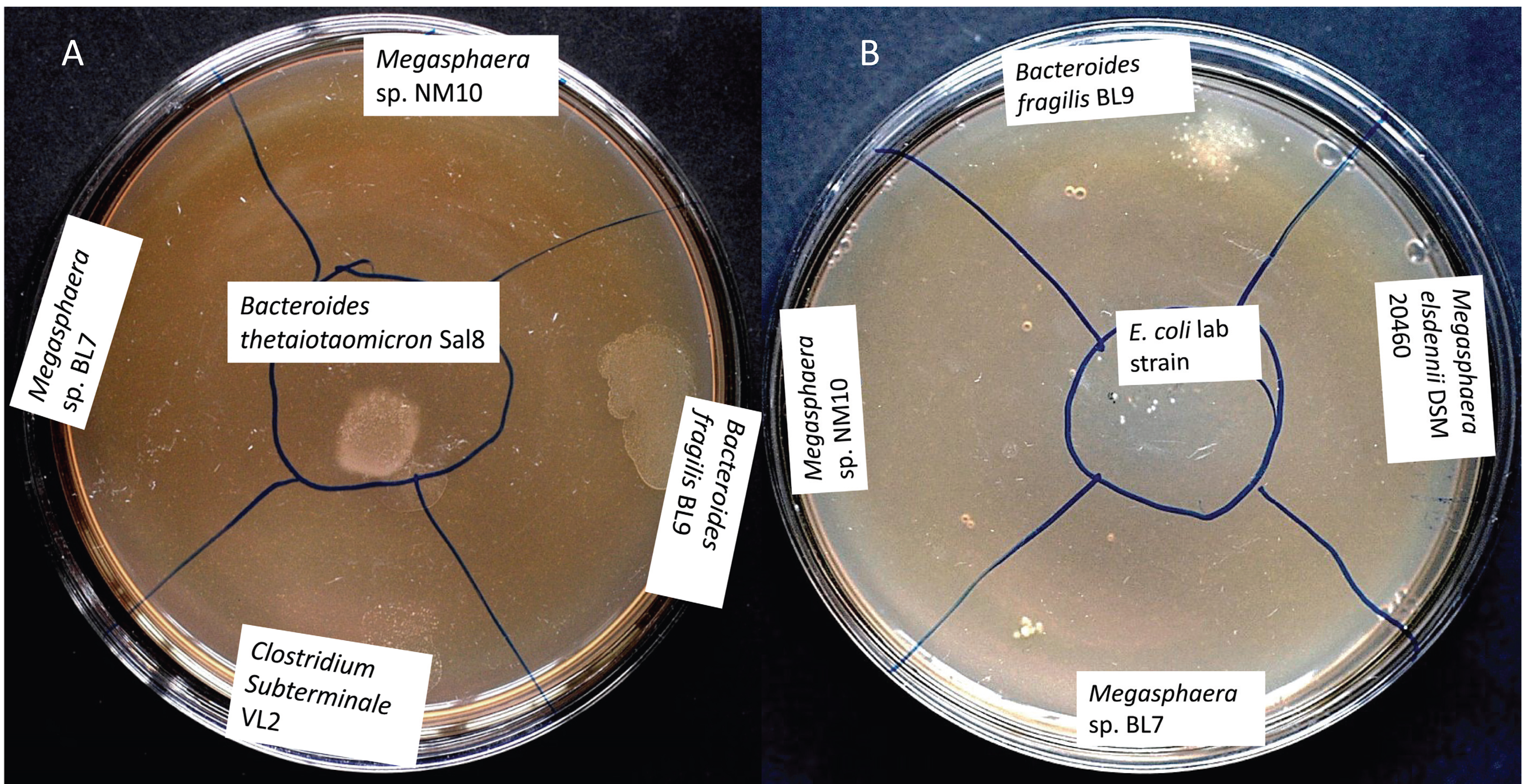

Supplement: Figure S1 — The plate representing absence of bile salt hydrolase (BSH) activity in the isolates Megasphaera sp. NM10, BL7 and M. elsdenii. The white zone of precipitation represents a positive result. A) Represents the absence of BSH activity in isolate NM10 and BL7, while presence of activity in the other isolates from the study. A) Represents the absence of BSH activity in isolate NM10, BL7 and M. elsdenii, E. coli is used a negative control. (TIF) [file pone.0079353.s001.tif]
